# Prevention or treatment of bronchial carcinoma: a literature review

**DOI:** 10.1186/1753-6561-6-S4-P18

**Published:** 2012-07-09

**Authors:** BL Green

**Affiliations:** 1 University of Leeds, UK

## Introduction

Bronchial carcinoma is the second most commonly diagnosed cancer, and is currently the number one cause of cancer death in the UK. Smoking is the major risk factor for the development of bronchial carcinoma and trends in smoking habits are reflected in incidence rates. Poor survival is due in part, to late development of symptoms, and a tendency for the presence of metastatic spread at presentation. This study aims to evaluate the efficacy of current treatments and whether or not prevention strategies should play a more prominent role in the future.

## Methods

Studies were selected for review from the electronic databases Medline and Pubmed, based on keyword search terms. Articles were then screened by title and abstract to assess their suitability for inclusion. Selected article references were screened manually for further source material. All articles were limited by English language and publication date post 1995.

## Results

Key studies indicate that treatment for limited stage disease is often curative, however over 60% of patients present with stage III or IV disease, and therefore treatment in this group is often palliative. Preventative measures such as the smoking ban have not yet been proven to reduce incidence rates, although studies have shown decreased levels of environmental carcinogens since the ban. Chemoprevention studies utilising nutritional supplementation identified selenium as reducing the relative risk of developing bronchial carcinoma. Furthermore, whilst screening programmes have shown earlier detection rates, this has not decreased associated mortality.

## Conclusions

The review suggests that current treatments are often limited by the extent of the disease process, and as such, are largely palliative. Late presentation and early metastases account for this situation and although there is limited data, current evidence suggests that screening programmes may not show any benefit in terms of mortality. It is therefore evident that more needs to be done to help prevent the development of bronchial carcinoma. Although preventative measures such as the smoking ban are likely to reduce mortality, this is evidently a long-term solution and interim measures, such as chemoprevention, should be considered in high risk groups.

